# Bio‐Inspired Gradient Heterogeneous Architectures for Ultrasensitive and Robust Strain Sensors via Programmable Crack Engineering

**DOI:** 10.1002/advs.77979

**Published:** 2026-09-27

**Authors:** Qi Wu, Tiantong Wang, Yinhao Wang, Yanggang Feng, Yan Huang

**Affiliations:** ^1^ School of Mechatronics Engineering Beijing Institute of Technology Beijing China; ^2^ School of Mechanical Engineering and Automation Beihang University Beijing China

**Keywords:** biomimetic sensors, crack‐based morphologies, flexible strain sensors, heterogeneous architectures, human‐machine interfaces, wearable medical devices

## Abstract

The development of flexible strain sensors is crucial for next‐generation wearable medical devices and human‐machine interfaces (HMIs). While conductive networks based on random microcracks have been widely employed to enhance sensor responsiveness, they are often limited by trade‐offs between sensitivity and measurement range. Inspired by the stress concentration mechanism in insect campaniform sensilla (CS), this study proposes a biomimetic heterogeneous bilayer strain sensor based on a deterministic macrostructure. The sensor employs a bilayer cladding architecture with a U‐shaped cross‐section and introduces a strain‐dependent path switching (SDPS) strategy by modulating the conductivity gradient between the low‐resistance core and the high‐resistance cladding layer. Through this structure, the sensor achieves an ultra‐high gauge factor (GF) of 301799, featuring an extremely low detection limit (0.067%) and excellent cycling stability (>11 000 cycles). By programmatically designing the crack geometry, the sensor demonstrates highly tunable sensitivity and range. Based on its exceptional performance, the sensor has been successfully applied to the monitoring of full‐scale human physiological signals and integrated into a high‐fidelity teleoperation system. This research not only provides a universal biomimetic paradigm for the design of high‐performance flexible sensors but also lays the theoretical foundation for the industrial application of precision medicine and intelligent robotic skin.

## Graphical abstract

Inspired by the insect campaniform sensillum, a heterogeneous bilayer strain sensor is developed based on a unique impedance mismatch and three‐dimensional structural dynamic percolation strategy. The sensor enables real‐time multi‐joint teleoperation of three joints and the gripper using a six‐degree‐of‐freedom robotic‐arm platform.

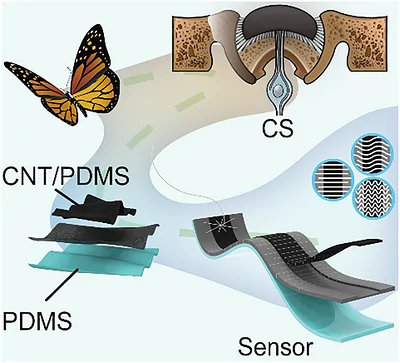

## 1 Introduction

Reliable acquisition and intelligent interpretation of physical signals underpin emerging intelligent perception, human‐machine interaction (HMI), and robotic systems. Recent studies exemplify the evolution of sensing platforms beyond signal acquisition toward task‐oriented intelligent perception, including bioinspired electrostatic‐field sensing for noncontact material perception and robotic control [1], large‐scale flexible arrays with low‐power in‐sensor intelligence [2], and machine‐learning‐assisted sensing interfaces for crop recognition [3] and user identification [4]. Within this broad landscape, flexible and stretchable strain sensors have emerged as pivotal components in physiological monitoring, HMI, and soft robotics owing to their exceptional conformability, lightweight configuration, and highly integrated architecture [5, 6, 7, 8, 9]. Driven by the evolution of wearable electronics, increasing research has focused on the dual imperatives of ultra‐high sensitivity and long‐term stability. However, contemporary material systems and structural designs often suffer from an intrinsic trade‐off between high sensitivity and mechanical robustness. Moreover, the fabrication of high‐performance sensors frequently necessitates sophisticated and costly laboratory environments, which impedes their scalable manufacturing and practical applications [10, 11, 12].

Based on structural characteristics and sensing mechanisms, flexible strain sensors can be categorized into three representative paradigms. The first category consists of sensors based on fiber and textile architectures. These typically leverage techniques such as electrospinning [13] and wet‐spinning [14] to incorporate conductive fillers into elastic polymer matrices. Given their one‐dimensional architecture, these sensors exhibit superior flexibility, extreme stretchability, and outstanding cycling stability [15, 16, 17]. However, since the internal conductive network of the fiber is tightly encapsulated by the elastic substrate, changes in resistance during the low‐strain phase are primarily governed by geometric deformation effects, resulting in a lower initial gauge factor (GF); furthermore, the non‐uniformity of Poisson's contraction reduces linearity. The second category involves sensors based on stochastic microstructures, including microcracks [6, 18, 19, 20, 21, 22, 23, 24], wrinkles [25], and microporous architectures [26]. These are typically fabricated via microstructured metal deposition [19], masked sputtering [6, 18, 20, 21], or thermal evaporation [22]. While these methods induce high‐sensitivity crack networks through prestressing, the stochastic nature of crack initiation severely constrains the linear range. Under cyclic loading, uncontrolled crack propagation leads to significant hysteresis and nonlinear fluctuations. Furthermore, reliance on high‐vacuum or complex masking techniques incurs high fabrication costs. The third category encompasses sensors with deterministic macro‐architectures. These achieve pre‐designed crack patterns through stencil transfer [27], screen printing [28], three‐dimensional (3D) printing [29], or precision mechanical incising, with serpentine crack structures [30, 31, 32, 33, 34, 35] being particularly representative. Such predefined geometric paths grant these sensors exceptional robustness during deformation. Nevertheless, as these structures typically operate on a macroscopic scale and lack effective internal signal amplification mechanisms, their GF often remains constrained, hindering their capability in detecting subtle physiological signals. Achieving microstructure‐level signal amplification within deterministic macro‐architectures, while preserving predictable crack propagation, remains a crucial challenge for next‐generation flexible strain sensors.

In nature, the campaniform sensillum (CS) in insects provides an exceptional biological model for resolving the aforementioned contradiction [36, 37, 38, 39]. As a sophisticated proprioceptive device, CSs are distributed throughout key regions of insects, including the legs, wings, and antennae. The core structure of the CS consists of a highly rigid cap and an extremely flexible joint membrane [40, 41]. When strain occurs, due to a significant modulus mismatch, the stress flow cannot penetrate the rigid core and is forced to accumulate in the surrounding soft membrane, causing intense local deformation and stimulating mechanosensitive neurons. This process of converting weak, widespread background strain into a localized, high‐intensity compression signal forms the physical basis for nonlinear signal amplification in biological systems.

Inspired by these biological mechanisms, this study designed a heterostructure sensor with a conductivity gradient and a 3D U‐shaped encapsulation topology. Unlike many conventional biomimetic sensors that primarily focus on morphological imitation, this study constructs a heterogeneous structure with high/low conductivity gradients. This design replicates the logic of local stiffness heterogeneity and energy flow guidance inherent in biological mechanoreceptors at the electrical level, thereby establishing a step‐like response mechanism. This design elevates the sensor from one‐dimensional geometric deformation detection to 3D topological path reconstruction. By regulating the concentration distribution of carbon nanotubes (CNTs), a conductivity gradient was created featuring a low‐resistance core and a high‐resistance sheath. This design implements a strain‐dependent path switching (SDPS) strategy: a predefined serpentine crack acts as a topological switch, forcibly redirecting the current path from the low‐resistance main channel to the high‐resistance bypass during deformation. This path‐reconstruction‐based sensing mechanism enables the sensor to achieve a sensitivity improvement across orders of magnitude. Furthermore, by predefining the geometric configuration of the crack, task‐oriented customization of sensitivity and linear range is achieved.

Through comprehensive electromechanical evaluations, the sensor demonstrated exceptional capabilities, achieving an ultra‐high sensitivity (GF = 301799), an extremely low detection limit (0.067%), and excellent cycling stability (>11 000 cycles). Benefiting from these superior characteristics, the sensor was successfully deployed in various practical scenarios, ranging from the continuous monitoring of full‐scale human physiological signals to integration within a high‐fidelity teleoperation system. Ultimately, this research provides a novel design paradigm for deterministic macro‐architectures in flexible electronics, paving the way for advanced applications in precision medicine and intelligent robotic skin.

## 2 Results and Discussion

### 2.1 Biomimetic Heterogeneous Architecture Design and Programmable Fabrication

**FIGURE 1:**
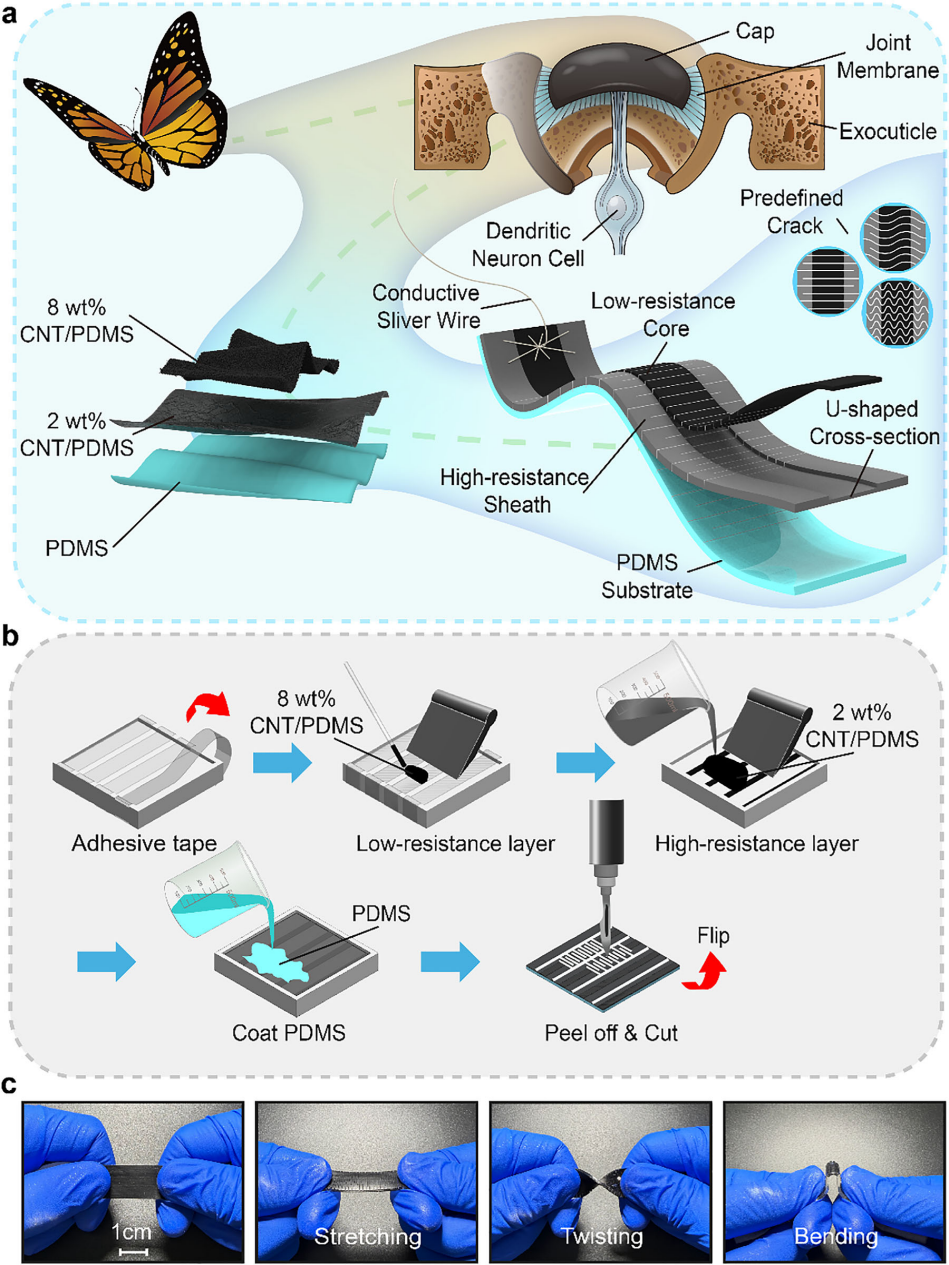
Design and fabrication of the biomimetic heterogeneous crack strain sensor. (a) The biomimetic design pathway, illustrating the biological inspiration from the insect campaniform sensillum (CS) anatomy, followed by an exploded view of the sensor's heterogeneous layers (8 and 2 wt.% CNT/PDMS, and pure PDMS), and the final assembled sensor schematic featuring the low‐resistance core, high‐resistance sheath, U‐shaped cross‐section, and conductive silver wires. The upper right inset shows three predefined crack geometries: linear, low‐curvature serpentine, and high‐curvature serpentine. (b) Schematic diagram of the sensor fabrication process. (c) Photographs demonstrating the mechanical compliance of the sensor under various deformation modes (stretching, bending, twisting).

In nature, the CS of insects exhibits exceptional sensitivity to strain, which stems from a sophisticated biomechanical transduction mechanism. As shown at the beginning of the biomimetic pathway in Figure 1a, CSs are widely distributed at the base of the wings and at the joints of the legs in insects such as butterflies, dragonflies, and bees. Anatomically, a CS comprises a rigid cap, collar, socket, and a joint membrane, and a mechanosensitive neuron containing a modified dendrite. Because the cap is extremely rigid while the joint membrane is extremely soft, stress cannot penetrate the rigid core and is instead forced to concentrate entirely on the surrounding soft tissue, causing severe localized deformation and stimulating mechanosensitive neurons. The core of this response lies in the stress flow control and energy accumulation resulting from localized stiffness inhomogeneities, a phenomenon known in biomechanics as stress concentration caused by modulus mismatch [36, 37, 38, 39, 40, 41].

Inspired by this biological paradigm, we developed a heterogeneous bilayer sensor featuring a programmed conductivity gradient. This design maps the stiff cap/soft membrane modulus‐step logic onto an electrical SDPS strategy based on impedance mismatch. Following the design pathway in Figure 1a, the structure of the sensor is progressively deconstructed from an exploded view of its constituent layers to the final assembled schematic. The sensitive layer comprises a central low‐resistance core (7 mm wide, 8 wt.% CNTs) and high‐sensitivity sheaths on both sides (4.5 mm wide, 2 wt.% CNTs). Under equilibrium, the low‐resistance core functions as the primary signal transmission channel, mimicking the biological rigid cap. The high‐resistance coating with a U‐shaped profile mimics a flexible joint membrane, ensuring not only electrical continuity in 3D space but also serving as a critical vertical electrical bypass. The introduction of this heterogeneous impedance gradient establishes the physical basis for current to escape from the low‐resistance core to the high‐resistance sheath under strain excitation. Furthermore, as highlighted in the inset of Figure 1a, by adjusting the geometric parameters of the predefined cracks (e.g., linear, low‐curvature serpentine, high‐curvature serpentine), it is possible to meet the customized requirements for GF and linear range in various application scenarios. Current is transmitted via conductive silver wires at both ends of the sensor.

The basic fabrication process of the sensor is shown in Figure 1b. The geometric boundaries of the central low‐resistance region are defined using the grooves of a polytetrafluoroethylene (PTFE) mold in combination with masking tape technology. The sensitive layer consists of a mixture of CNT and polydimethylsiloxane (PDMS). A high‐concentration (8 wt.%) CNT/PDMS mixture is thoroughly stirred and then blade‐coated into the gaps between the masking tapes; after heating and curing, the central low‐resistance region of the sensing layer is obtained. Subsequently, the masking tape is removed, and a low‐concentration (2 wt.%) CNT/PDMS mixture is uniformly applied via blade coating to fill the entire grooved area of the PTFE mold, ultimately forming a bilayer heterogeneous sensing layer with a U‐shaped encapsulation feature. Finally, the PDMS substrate is poured in, cured, and demolded. A serpentine zigzag structure is then cut into the surface of the sensitive layer using a mechanical cutting machine to create the predefined cracks mentioned above. Specific operational steps are shown in Figure S1. Thanks to the excellent compatibility between the all‐carbon material system and the PDMS substrate, the fabricated sensor exhibits outstanding mechanical compliance, maintaining signal stability under multimodal deformations such as stretching, bending, and torsion (Figure 1c).

### 2.2 Staged Structural Evolution and SDPS Mechanism

#### 2.2.1 Equivalent Circuit Modeling

**FIGURE 2:**
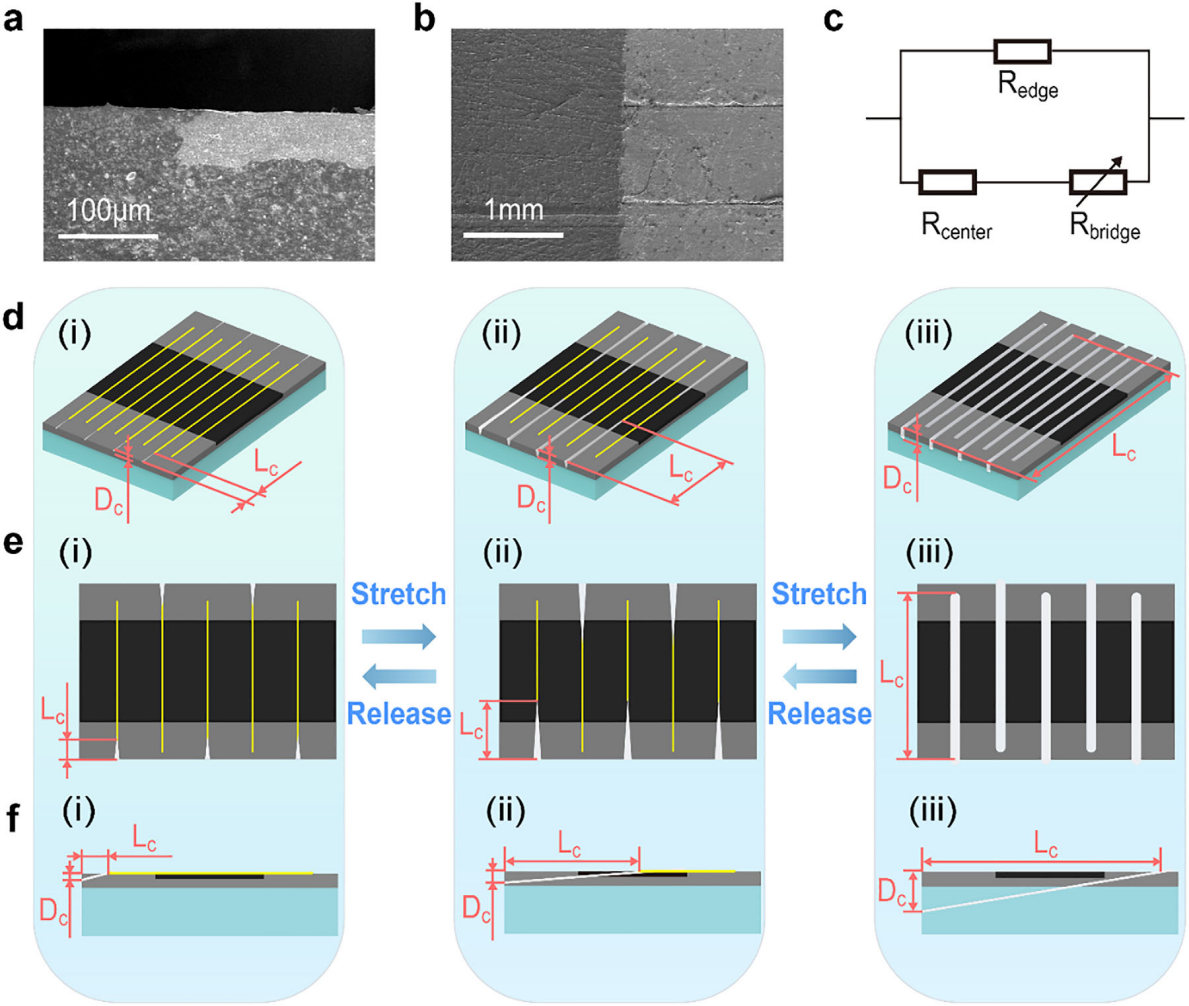
Microstructural characterization of the sensor and the mechanism of its structural dynamic evolution. (a, b) SEM images of key features of the sensor: (a) U‐shaped embedded cross‐section of the core–shell interface; (b) Top view of the crack morphology in the sensitive layer. (c) Equivalent circuit diagram of the SDPS sensor. (d–f) Stepwise evolution of the sensor during the stretching and relaxation processes, illustrating the transition from initial contact through progressive opening of the predefined cracks to complete interfacial separation: (d) 3D view, (e) top view, (f) cross‐section. (Note: The yellow lines indicate the locations of the predefined cracks before opening, while Lc and Dc denote the effective in‐plane opened length and through‐thickness separation depth, respectively).

The sensor's double‐layered nested architecture was clearly verified by cross‐sectional scanning electron microscopy (SEM) images (Figure 2a). High‐contrast secondary electron signals revealed that the low‐resistance core, composed of a high concentration of CNT/PDMS, was completely nested within a low‐concentration, highly elastic, high‐resistance sheath. Figure S2a clearly shows the extension of the central concave region onto the surface, with an overall very smooth profile. Figure 2b displays the top‐view morphology of this heterogeneous sensing layer. In the figure, the light‐colored, brittle, low‐resistance core exhibits a distinct array of mechanically predefined linear cracks, while the PDMS‐rich, high‐resistance sheath, due to its lower Young's modulus and higher toughness, shows less pronounced cracking. Figure S2c reveals the internal morphology of the low‐resistance region under high‐magnification electron microscopy; cracks are also visible in the high‐resistance region when magnified (Figure S2d). The low‐resistance central core provides excellent initial conductivity, ensuring low noise under static conditions; in steady‐state operation, current is primarily concentrated in the low‐resistance central core. Once tensile loading progressively opens the predefined serpentine cracks, the dominant low‐resistance pathway is interrupted, forcing the current to switch from a low‐resistance path to a high‐resistance path. Due to the significant contrast in resistivity between the U‐shaped sheath and the central core, this path‐reconstruction‐based sensing mechanism achieves nonlinear amplification of weak signals.

The sensor's operating principle can be modeled as a series of parallel resistive units, as shown in Figure 2c. Each unit consists of a high‐resistance detour branch (R_edge_, representing the serpentine path along the high‐resistance edge) and a low‐resistance shortcut branch. The shortcut branch is formed by the series connection of the core resistance (R_center_) and the strain‐sensitive vertical bridge resistance (R_bridge_). The total resistance is expressed as:

(1) $$R_{total}\left(\varepsilon \right)=N\cdot {\left(\frac{1}{R_{edge}}+\frac{1}{R_{center}+R_{bridge}\left(\varepsilon \right)}\right)}^{-1}$$

where N is the number of serpentine elements, and ε represents the applied strain. As strain is applied, R_bridge_ undergoes a nonlinear surge, regulating the redistribution of current between the low‐impedance shortcut and high‐impedance detour branches.

#### 2.2.2 Progressive Opening of Predefined Cracks

The sensing behavior of the sensor is governed by a coupled opening process spanning both the 3D longitudinal cross‐section and the two‐dimensional macroscopic plane (Figure 2d–f). At the microscopic scale, through‐thickness opening occurs rapidly: owing to the extremely thin sensing layer (≈ 50 µm), tensile loading rapidly reduces direct electrical contact across the predefined crack interface in the first‐layer core, thereby activating the R_bridge_ component. Unlike traditional single‐component crack sensors, this structure utilizes a 3D vertical shunting mechanism, involving a dimensional transition from horizontal surface transmission to vertical deep penetration (Figure 2f). As separation progresses through the thickness along the predefined crack interface, the effective opening depth D_c_ increases, forcing the current to penetrate deeper into the high‐resistance region of the second layer. At the macroscopic scale, in‐plane opening along the predefined crack path evolves more gradually, as shown in the top‐view schematic in Figure 2e. As strain increases, following the rapid loss of through‐thickness contact, progressive in‐plane opening along the predefined crack dominates the subsequent response. The effective opened length L_c_ along the predefined crack gradually increases, and the topological function of the U‐shaped structure begins to manifest: the current is confined within a narrow shunt path, and lateral contraction (Poisson effect) causes the effective conductive area A_eff_ to decrease sharply. Combining Ohm's law and the Simmons tunneling model, the evolution equation for R_bridge_ is:

(2) $$R_{bridge}\left(\varepsilon \right)=\rho_{trans}\cdot \frac{L_{eff}\left(\varepsilon \right)}{A_{eff}\left(\varepsilon \right)}\cdot exp\left(\beta \cdot \Delta d\right)$$

Here, ρ_trans_ represents the equivalent transition resistivity at the heterojunction interface, β is the tunneling sensitivity factor, and Δd is the tunneling gap that increases with strain. This equation captures the deep coupling between geometric path reconstruction and the quantum tunneling effect. As the crack opens fully in the longitudinal direction (D_c_ reaches its maximum value), the opened portion along the predefined crack increases in plane, the cross‐sectional area narrows, and A_eff_ decreases sharply. Meanwhile, as the crack gap widens, the tunneling effect weakens, and exp(βΔd) increases sharply, leading to a massive rise in R_bridge_ and resulting in a significant change in resistance.

#### 2.2.3 Phased Electromechanical Response

Based on the aforementioned mechanism, the sensor response is divided into three phases:

##### 2.2.3.1 Virtual Short‐Circuit Mode (ε = 0)

Interfacial adhesion maintains physical contact between the opposing faces of the predefined crack in the initial state. Due to the direct physical contact of the first layer of the core, the vertical bridging resistance R_bridge_ can be neglected. Current flows primarily through the R_center_ branch (R_center_ << R_edge_), resulting in an extremely low initial resistance (R_0_). This virtual short‐circuit mode significantly suppresses thermal noise, improves the signal‐to‐noise ratio (SNR) for weak signal detection, and establishes a low baseline for calculating high GF.

##### 2.2.3.2 Transition From Vertical Bridging to Tunneling (0< ε < ε_sat_)

As shown in stages (i) and (ii) of Figure 2d–f, the crack exhibits V‐shaped separation as tensile loading begins. Direct electrical contact across the predefined crack in the first‐layer core is lost first, blocking the direct surface pathway. The current does not experience a sudden open‐circuit failure but is forced to flow vertically into the underlying second layer. This generates a transition resistance R_bridge_(ε), which exhibits a steep increase due to the combined effects of the growing tunnel distance and the abrupt decrease in A_eff_ caused by the Poisson effect. This stage is the primary source of the ultra‐high GF.

##### 2.2.3.3 Topological Saturation Mode (ε ≥ ε_sat_)

As shown in stage (iii) of Figure 2d–f, when the strain exceeds the saturation threshold, the predefined crack interface approaches complete opening and A_eff_ approaches 0, causing R_bridge_ to approach infinity. The low‐resistance shortcut branch is electrically disconnected, and the current path undergoes a topological reconfiguration, flowing entirely through the high‐resistance detour branch (R_edge_). At this point, the sensor's performance is governed by the geometric deformation of the high‐resistance edge, exhibiting a stable linear response and preventing electrical failure of the device under extreme strain.

### 2.3 Performance Customization via Geometric Regulation and Finite Element Analysis

**FIGURE 3:**
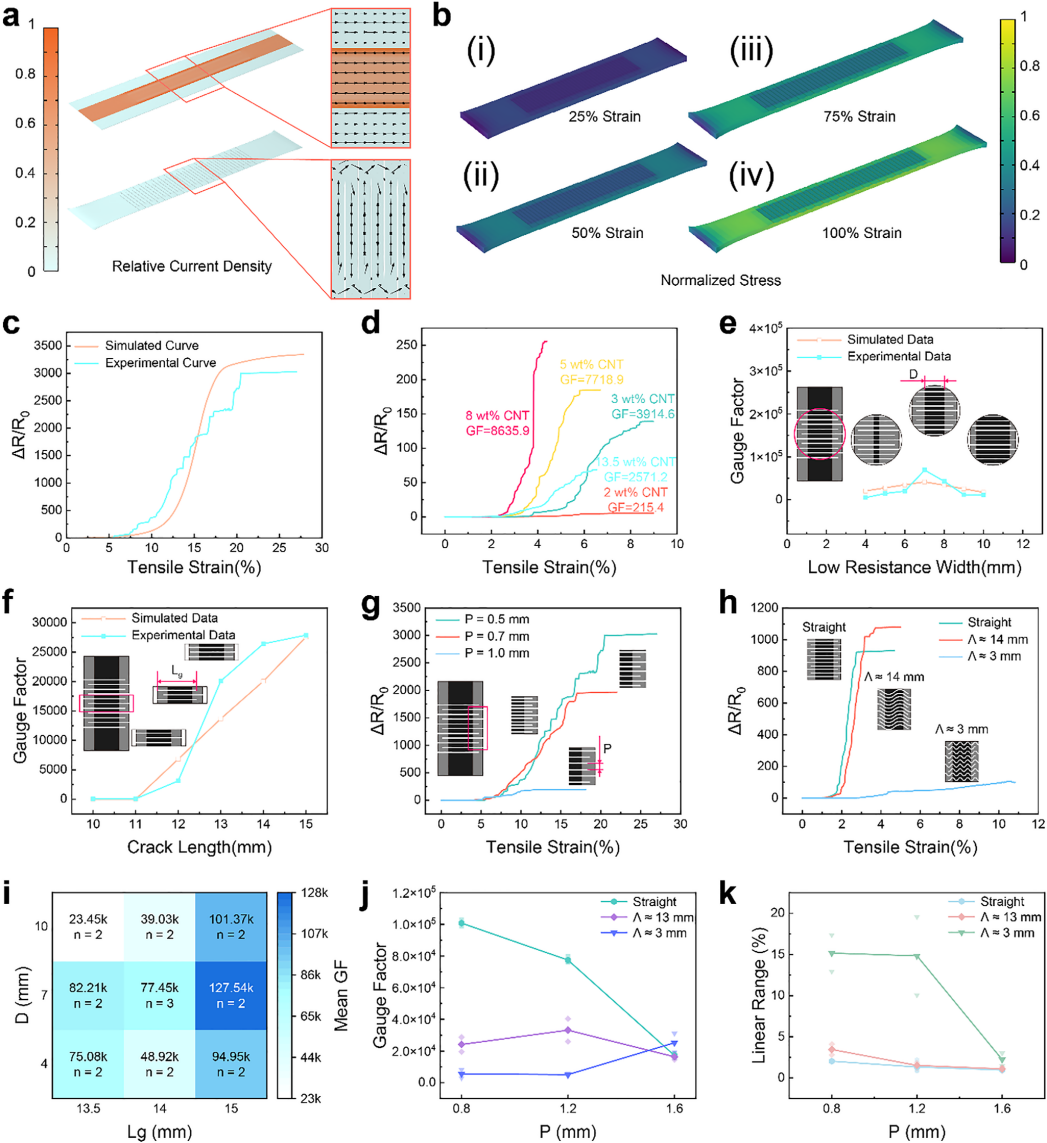
Mechanistic verification and geometry‐enabled performance customization of the SDPS sensor. (a) FEA simulations showing the relative current density distribution (normalized from 0 to 1) in 3D models. The top and bottom panels represent the unstretched and completely stretched states, respectively. The magnified views on the right incorporate volume arrows to visualize the local current paths, illustrating the transition from straight current transmission to a lateral detour (electrical bypass) mechanism upon crack opening. (b) 3D maps of the normalized stress distribution (scaled from 0 to 1) highlighting stress concentration at four consecutive stretching stages: (i) 25%, (ii) 50%, (iii) 75%, and (iv) 100% of the maximum applied displacement. (c) Comparison between the experimental (Exp.) and simulated (Sim.) relative resistance change (ΔR/R_0_) as a function of tensile strain. (d) Sensitivity comparison of single‐layer sensors with different CNT concentrations. (e, f) Comparison of experimental (Exp.) and simulated (Sim.) GF with respect to (e) the width of the central conductive core, D, and (f) the crack length, L_g_. (g, h) Experimental GF curves demonstrating the tunability of sensing performance by adjusting (g) crack pitch, P, and (h) crack geometry (straight vs. curved). (i) Heatmap of the mean GF obtained from the D × L_g_ factorial experiment; the number of independently fabricated devices is indicated in each cell. (j, k) Interaction plots showing the effects of crack pitch, P, and crack‐path geometry on (j) GF and (k) linear sensing range. Light symbols represent individual devices, whereas connected solid symbols represent the mean. Unless otherwise specified, *n* = 2 independently fabricated devices per condition; the shared reference condition (D = 7 mm, L_g_ = 14 mm, P = 1.2 mm, straight crack) contained n = 3 devices.

To further elucidate the mechanism of SDPS, we performed a finite element analysis (FEA) of the relative current density and normalized stress distributions during the sensor's stretching process (Figure 3a,b). In the model, the crack paths were prescribed by the deterministic cut geometry, and the loading process was represented by progressive separation and electrical contact loss along these predefined interfaces. In the initial, unstressed state (Figure 3a, top), due to a significant conductivity gradient between the core and the sheath, the current lines are highly concentrated in the low‐resistance core region (highlighted in orange on the 0 to 1 scale), exhibiting uniform seepage characteristics with straight current paths, as indicated by the volume arrows. When tensile strain is applied, progressively opening the predefined crack interfaces (Figure 3a, bottom), the local electric field undergoes a dramatic restructuring. FEA results show that progressive electrical contact loss across the crack interfaces suppresses transverse current transfer, causing the macroscopic relative current density to plummet to near zero (blue region) and forcing the current to switch from horizontal transmission to a lateral detour mode along the U‐shaped structure. The magnified views (Figure 3a, right) explicitly visualize this detour mechanism via the diverted volume arrows.

The mechanical driving force behind this electrical impedance switching is further revealed by the 3D normalized stress maps (Figure 3b). Tracked at four loading levels corresponding to 25%, 50%, 75%, and 100% of the maximum applied displacement (Figure 3bi–iv), the stress (scaled from 0 to 1) is shown to severely concentrate at the pre‐defined crack regions, dominating the sequential opening behavior. As the ultimate manifestation of these coupled electromechanical behaviors, Figure 3c demonstrates an excellent match between the experimental and simulated relative resistance change (ΔR/R_0_) curves as a function of tensile strain, providing a solid theoretical explanation for the sensor's high GF.

#### 2.3.1 Optimization of Material Composition

The initial resistance and sensing sensitivity of the sensor are governed by the synergistic interaction between filler concentration and geometric dimensions. Experimental results (Figure S3) indicate that the resistivity (Ω·m) of the CNT/PDMS composite exhibits a typical nonlinear decrease with increasing mass fraction, crosses the percolation threshold within the range of 1 to 4 wt.%, and stabilizes near 8 wt.%. Figure 3d shows a comparison of the sensitivity of monolayer sensors with different CNT contents. The results indicate that as the concentration increases, the GF first increases and then decreases, with a peak occurring near 8 wt.%. However, the sensitivity of single‐component sensors is limited by the traditional grid breakage mechanism, making it difficult to achieve a breakthrough in performance. In this study, by constructing a heterogeneous combination of 8 and 2 wt.%, and utilizing maximized impedance contrast to drive the SDPS process, the sensor GF was significantly enhanced. The selection of a 2 to 8 wt.% ratio not only ensures an extremely high SNR and low baseline noise but also effectively suppresses filler agglomeration at high concentrations by optimizing the rheological properties of the slurry, thereby ensuring consistent device fabrication.

The proposed heterogeneous two‐layer sensor architecture provides a universal platform for performance customization. As shown in Figure S4, GF, linear range, and robustness can be precisely programmed by adjusting the following key geometric parameters:

#### 2.3.2 Center Low‐Resistance Width (D)

The width (D) of the central low‐resistance region governs the fundamental trade‐off between the initial resistance R_0_ and the saturation resistance R_final_. As shown in Figure 3e, both finite element simulation results and experimental data exhibit distinct convex curves, with peak sensitivity occurring at D ≈ 7 mm. When D is too narrow, the initial channel impedance becomes excessively high; when D exceeds a critical threshold (approximately 7 mm), a current shunting effect occurs. The widened low‐resistance region spatially overlaps with the high‐stress bottleneck region, thereby partially shielding the high‐sensitivity area and leading to a decline in the sensor's overall performance. This optimal width ensures the smallest possible R_0_ while preserving the integrity of the high‐resistance switching pathway.

#### 2.3.3 Crack Length (L_g_)

The dependence of GF on crack length follows the stress amplification logic in fracture mechanics. The crack length must exceed the intermediate width (i.e., L_g_ > D) to physically sever the low‐resistance core, thereby triggering the SDPS mechanism. As the crack length L_g_ increases, the width of the remaining connecting bridge (W_bridge_ = W_sensor_ – L_g_) narrows. Based on Inglis's fracture mechanics theory, the stress concentration factor (K_t_) at the bridge neck increases proportionally with crack length, converting macroscopic strain into severe local deformation:

(3) $$K_{t}\approx 1+2\sqrt{\frac{L_{g}}{r_{tip}}}$$

here, r_tip_ represents the radius of the crack tip. The amplified local strain drives a sharp increase in the bridging resistance, resulting in a superlinear dependence of the GF on L_g_. As shown in the following equation:

(4) $$GF\left(L_{g}\right)\propto exp\left(\sqrt{L_{g}}\right)$$

This indicates that maximizing crack length while maintaining structural integrity is the most effective strategy for achieving ultra‐high GF; simulations and experiments also show the same trend, as illustrated in Figure 3f. Finally, to ensure the sensor's cyclic robustness, a longer crack length of 14 mm was selected as the final sensor crack length, leaving approximately 2 mm of redundancy.

#### 2.3.4 Crack Pitch (P)

The total resistance change of the sensor is the cumulative sum of the tunnel resistance changes in each unit. For a straight crack array with a fixed active length, decreasing the crack pitch increases the number of predefined electrically active crack units. The resistance changes associated with these units accumulate during stretching, resulting in a larger macroscopic

ΔR/R_0_, thereby achieving higher GF. Experiments and simulations indicate (Figure 3g) that the slope of the resistance change rate (i.e., GF) increases significantly as the crack pitch decreases. Here, P is defined as the center‐to‐center pitch between two adjacent predefined cuts, with a unit of mm. However, the crack pitch also controls the spacing and mechanical interaction between neighboring crack‐opening regions. Therefore, its influence cannot be assumed to remain unchanged for wavy cracks, whose deformation involves additional bending and geometric unfolding. The geometry‐dependent effect of crack pitch is examined through the targeted factorial experiment in Section 2.3.6.

#### 2.3.5 Crack Topological Geometry

By introducing a geometric tortuosity factor (λ = L_path_/L_g_), the sensor's response mode can be optimized for different application scenarios (Figure 3h). Here, L_path_ is the total actual path length of the crack along the wavy line, and L_g_ represents the straight‐line distance between the two endpoints of the crack, and Λ represents the period of the wave, i.e., the distance between two wave crests. Specifically, the linear topology (λ = 1) produces a sharp path interruption even under minimal strain, providing maximum GF for detecting weak signals. It is suitable for detecting faint physiological signals where ultimate sensitivity is sought, but its linear range is limited.

2.3.5.1

*Serpentine Topology (λ > 1)*: Increasing the wave frequency (density) enhances the tortuosity.

Since the curved geometric structure can alleviate local stress concentrations through bending deformation during stretching, it delays the process of complete path interruption. Compared to the step response of linear cracks, this significantly broadens the linear sensing range and improves the linearity of the sensitivity curve, making it suitable for the precise capture of large‐scale movements. Because the extent of this unfolding also depends on the spacing between adjacent cracks, crack pitch and crack‐path geometry are expected to exhibit a coupled effect, which is evaluated in Section 2.3.6. Furthermore, in addition to the elastic recovery of the substrate, the serpentine crack introduces additional geometric elastic potential energy, effectively overcoming microscopic friction and adhesion between crack surfaces. This enhances recovery capability, thereby significantly reducing electromechanical hysteresis and improving dynamic response speed.

#### 2.3.6 Targeted Combinatorial Analysis of Geometric Parameters

The preceding one‐factor studies established the individual response trends associated with D, L_g_, P, and crack‐path geometry. However, a one‐factor‐at‐a‐time analysis cannot determine whether the effect of one parameter depends on another. We therefore performed two targeted 3 × 3 full‐factorial experiments to examine the most physically plausible parameter dependencies. The D × L_g_ experiment evaluated the combined regulation of low‐resistance‐core interruption and the remaining conductive bridge, while the P × geometry experiment evaluated the competition between crack number, crack spacing, and topology‐dependent opening. In the D × L_g_ experiment, P was fixed at 1.2 mm and a straight crack geometry was used, with D = 4, 7, and 10 mm and L_g_ = 13.5, 14, and 15 mm. In the P × geometry experiment, D and Lg were fixed at 7 and 14 mm, respectively, while P = 0.8, 1.2, and 1.6 mm was combined with straight, large‐period wavy (Λ ≈ 13 mm), and small‐period wavy e(Λ ≈ 3 mm) cracks. These levels bracketed the reference geometry and the experimentally accessible ranges identified in the preceding single‐factor experiments. Each condition included two independently fabricated devices, except the shared reference condition (D = 7 mm, L_g_ = 14 mm, P = 1.2 mm, straight crack), for which three devices were tested. Exploratory Type III two‐factor analysis of variance (ANOVA) was performed on the log10‐transformed device‐level GF and linear‐range values. The complete statistical results are summarized in Table S2.

The D × L_g_ heatmap in Figure 3i shows that the largest mean GF was obtained at D = 7 mm and L_g_ = 15 mm (approximately 1.28 × 10^5^). Exploratory Type III two‐factor ANOVA performed on the log10‐transformed device‐level GF values identified significant main effects of D (F(2,10) = 5.75, p = 0.0217) and L_g_ (F(2,10) = 6.00, p = 0.0194), whereas the D × L_g_ interaction did not reach statistical significance (F(4,10) = 2.00, p = 0.170). Thus, within the investigated parameter space, D and L_g_ primarily provide additive rather than strongly non‐additive control over GF. The corresponding D × L_g_ interaction plots for GF and linear sensing range, including the individual‐device values, are provided in Figure S5. No statistically significant main or interaction effects were identified for the linear sensing range in the D × L_g_ experiment; the complete ANOVA results are provided in Table S2.

A markedly different behavior was observed in the P × geometry experiment (Figure 3j). For straight cracks, increasing P from 0.8 to 1.6 mm substantially decreased GF. In contrast, the large‐period wavy geometry exhibited its highest mean GF at the intermediate pitch, whereas the small‐period wavy geometry showed low GF at P = 0.8 and 1.2 mm but a pronounced increase at P = 1.6 mm. Accordingly, the main effect of crack geometry (F(2,10) = 56.87, *p* < 0.0001) and the P × geometry interaction (F(4,10) = 20.66, *p* < 0.0001) were significant, whereas the averaged main effect of P was not (F(2,10) = 0.84, p = 0.461). These results demonstrate that crack pitch cannot be interpreted independently of crack‐path geometry.

The interaction was also reflected in the linear sensing range (Figure 3k). The small‐period wavy geometry provided a mean linear range of approximately 15% at P = 0.8 and 1.2 mm, substantially exceeding those of the straight and large‐period wavy cracks, although at the expense of GF. Significant effects were obtained for P (F(2,10) = 19.22, p = 0.0004), geometry (F(2,10) = 40.73, *p* < 0.0001), and their interaction (F(4,10) = 3.61, p = 0.0454).

Taken together, these results translate the factorial analysis into practical design rules. Within the D × L_g_ design, D and L_g_ primarily regulate GF without a statistically resolved interaction, whereas within the P × geometry design, crack pitch must be selected jointly with crack‐path geometry because their effects on GF and linear sensing range are interdependent. Specifically, the straight‐crack configuration at P = 0.8 mm favors high sensitivity, whereas the small‐period wavy geometry at P = 0.8 – 1.2 mm favors an extended linear sensing range. This customization capability, enabled by deterministic cracking, therefore provides an experimentally supported route for task‐oriented sensor design, in which geometric combinations are selected according to application‐specific priorities rather than by maximizing a single parameter. Within the investigated parameter space, this framework supports the rational engineering of flexible strain sensors with application‐specific performance [42, 43, 44, 45, 46, 47, 48, 49, 50, 51, 52, 53, 54, 55, 56].

### 2.4 Electromechanical Performance, Manufacturing Reproducibility, and Robustness Under Harsh Conditions

**FIGURE 4:**
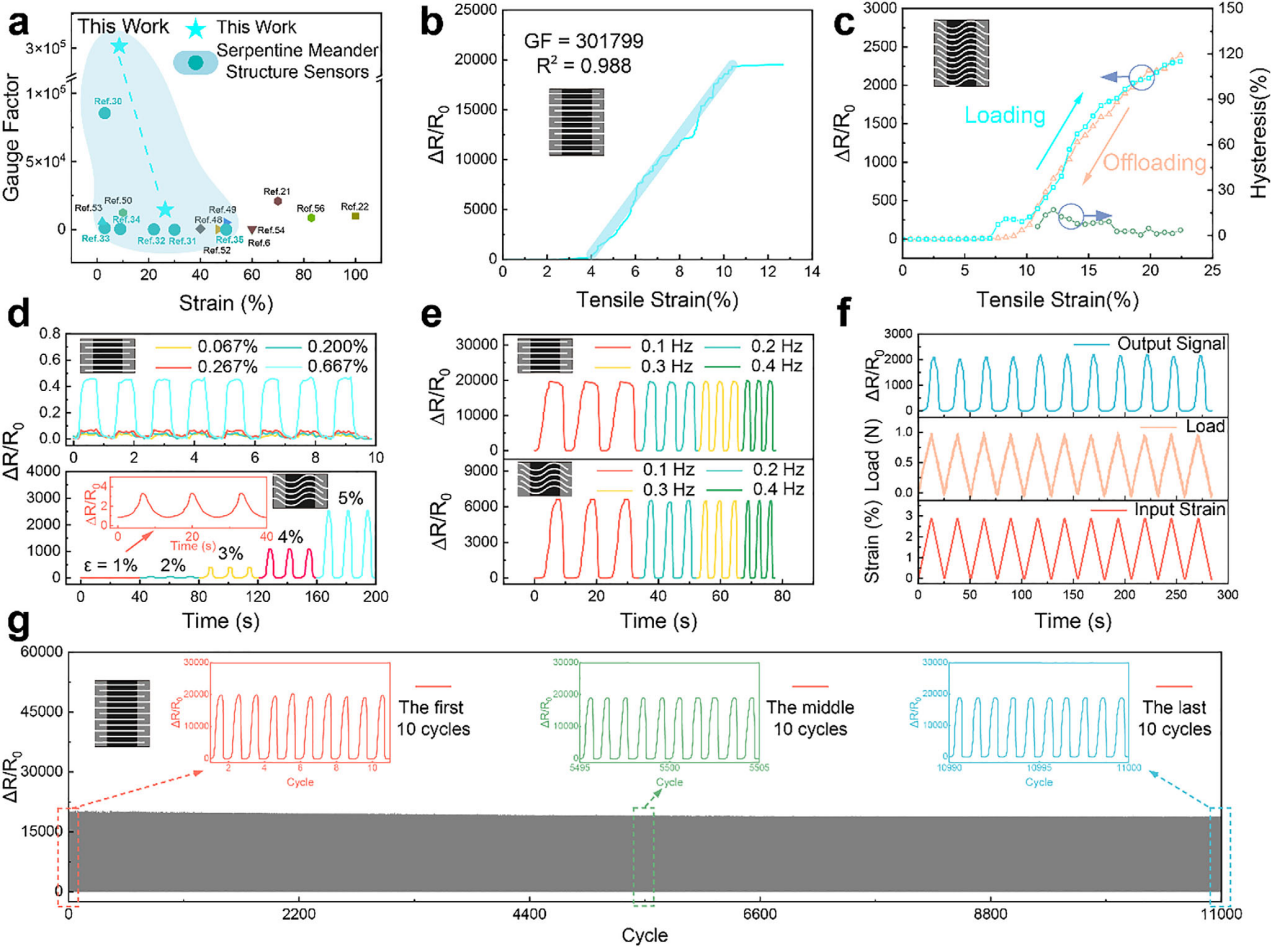
Characterization of the sensor's electromechanical properties. (a) Comparison of the GF and sensing range of the sensor developed in this work with those reported in the literature. (b) Relative resistance changes of the linear crack sensor, demonstrating an extremely high GF. (c) The hysteresis curve of the curved crack sensor demonstrates minimal hysteresis and high linearity. (d) Transient response of the sensors under step loads at small strains (top) and large strains (bottom). (e) Frequency response tests for linear (top) and curved (bottom) sensors (0.1–0.4 Hz). (f) Verification of the correspondence between the resistive response and displacement, force, and strain. (g) Long‐term electromechanical stability test of the linear crack sensor over 11 000 cycles.

We conducted a comprehensive comparison of the sensor's performance metrics with those of representative flexible strain sensors reported in recent studies (Figure 4a) [6, 21, 22, 30, 31, 32, 33, 34, 35, 48, 49, 50, 52, 53, 54, 56]. The experimental results (indicated by the blue pentagrams in the figure) demonstrate that the bio‐inspired heterogeneous sensor proposed in this study has successfully overcome the traditional trade‐off between GF and sensing range. Notably, compared to sensors with similar macroscopic serpentine structures (green circles in the figure), the sensor fabricated in this study achieved an order‐of‐magnitude breakthrough in GF, reaching the peak value currently known for sensors with deterministic macroscopic structures. Simultaneously, by predefining the crack shape, the sensor can accommodate an adjustable linear sensing range. This leap in performance is attributed to the significant impedance contrast provided by the heterogeneous bilayer architecture, as well as the nonlinear signal amplification effect driven by the SDPS strategy.

By precisely etching a linear crack, the sensor exhibits a resistance surge spanning multiple orders of magnitude under minimal strain (Figure 4b), with a GF value as high as 301799 while maintaining excellent linearity (R^2^ = 0.988). This demonstrates the absolute advantage of the heterogeneous current‐sharing mechanism in capturing weak signals. Meanwhile, the serpentine crack sensor, benefiting from the geometric elastic potential energy introduced by the serpentine topology, not only expands the linear sensing range but also exhibits minimal hysteresis error (Figure 4c), playing a crucial role in high‐fidelity HMI applications.

Under dynamic loading, the sensors demonstrate extremely high response frequencies and signal tracking capabilities. As shown in Figure 4d, the linear crack sensor exhibits exceptional transient recognition capability for minute strains as low as 0.067%. The serpentine crack sensor exhibits a stable step‐response plateau under cyclic loading at five strain amplitudes ranging from 1% to 5% large strain. Furthermore, during multi‐frequency cyclic testing at frequencies ranging from 0.1 to 0.4 Hz (Figure 4e), the output waveforms maintained a high degree of consistency. Figure 4f further validates the highly linear mapping relationship between the resistive response and the applied load (force, strain). In terms of long‐term reliability, the linear crack sensor maintained stable peak resistance values over 11 000 consecutive tensile cycles, with no significant baseline drift or performance degradation due to structural fatigue (Figure 4g), and the serpentine crack sensor exhibited the same cyclic stability (Figure S6). This not only validates the extremely strong interfacial bonding between the all‐carbon heterostructure and the PDMS substrate but also demonstrates the topological stability of the heterostructure under long‐term cyclic stress.

**FIGURE 5:**
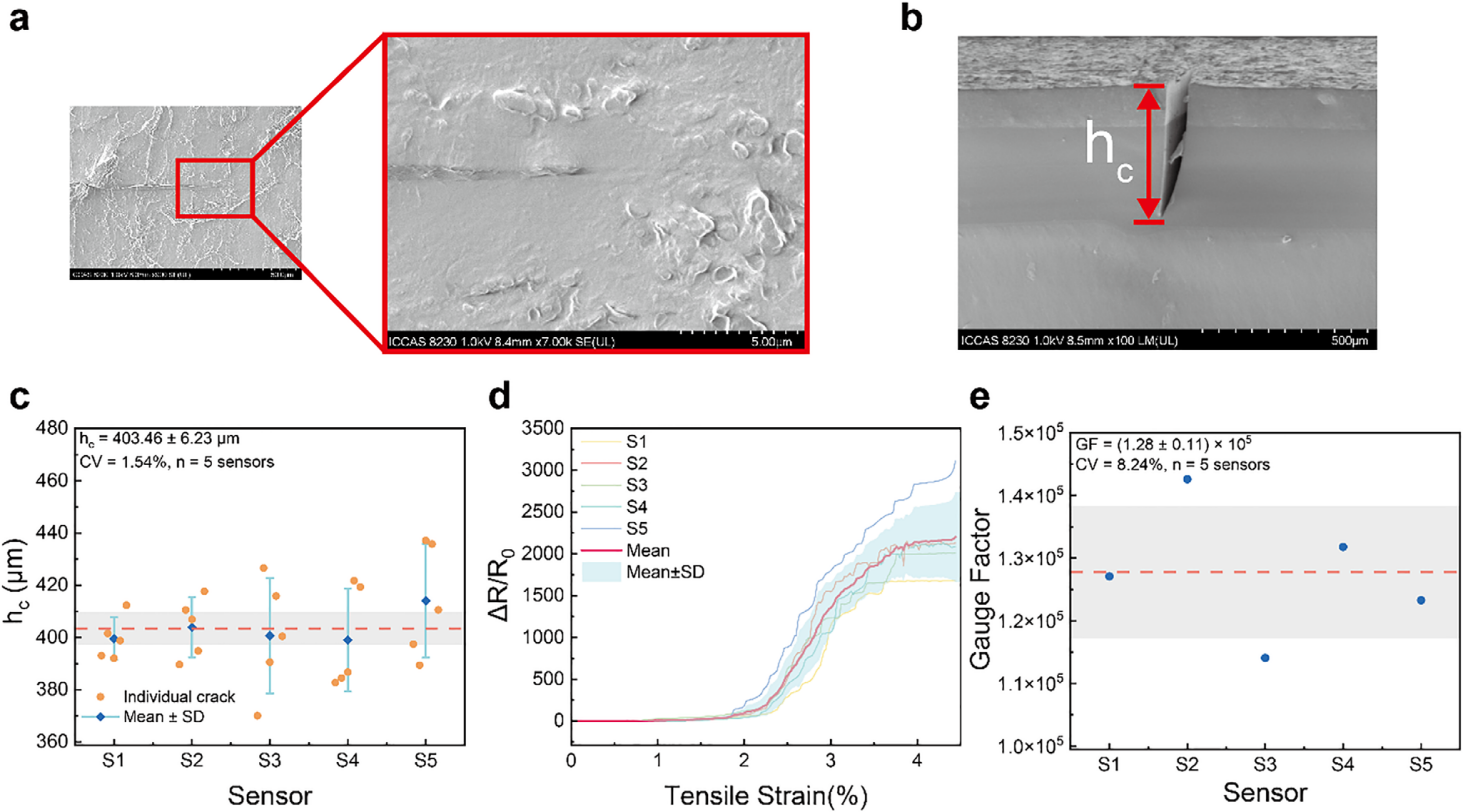
Geometric and electromechanical reproducibility of the predefined crack sensors. (a) Representative SEM images of a predefined crack at 900 × and 7000 × magnifications, showing the tightly closed crack morphology in the unloaded state. (b) Cross‐sectional SEM image illustrating the definition and measurement of the out‐of‐plane crack depth (h_c_). (c) Crack‐depth distributions obtained from five independently fabricated sensors, with five randomly selected cracks measured on each sensor. Orange circles represent individual crack‐depth measurements, while blue diamonds and error bars represent the sensor‐level mean and within‐sensor SD, respectively. The red dashed line and gray shaded region denote the overall mean and between‐sensor SD calculated from the five sensor‐level means. (d) Relative resistance changes of five independently fabricated sensors under tensile loading. The magenta curve and blue shaded region represent the pointwise mean and SD, respectively. (e) GF values of the five sensors. The red dashed line and gray shaded region indicate the mean and between‐sensor SD, respectively.

To further evaluate the manufacturing reproducibility of the predefined crack sensors, five independently fabricated sensors prepared using identical casting and mechanical cutting procedures were systematically characterized. Five cracks were randomly selected from each sensor and examined at 900 × magnification, providing a total of 25 low‐magnification SEM images (Figure S7). In addition, the C3 crack from each sensor was observed at 7000 × magnification to compare the high‐magnification crack morphologies among the five independently fabricated devices (Figure S8). A representative multiscale SEM observation is presented in Figure 5a. In the unloaded state, the opposing crack faces remained in close contact, resulting in an extremely narrow crack opening. Consequently, a stable boundary for quantitatively determining the in‐plane tip radius or opening angle could not be reliably segmented, even at high magnification. Because the apparent contrast may also be affected by specimen tilt, mounting‐induced height differences, and SEM shadowing, no potentially artifact‐prone numerical value was assigned to the in‐plane crack‐tip sharpness. These observations instead demonstrate the tightly closed initial state of the mechanically predefined cracks.

The out‐of‐plane crack depth, h_c_, was quantitatively determined from cross‐sectional SEM images, as illustrated in Figure 5b. Five randomly selected cracks were measured on each sensor, and the individual measurements together with the sensor‐level mean and within‐sensor standard deviation (SD) are presented in Figure 5c. The five independently fabricated sensors exhibited an overall mean crack depth of 403.46 ± 6.23 µm, corresponding to a between‐sensor coefficient of variation (CV) of 1.54% (*n* = 5 sensors; five cracks per sensor). The electromechanical reproducibility was further evaluated using the relative resistance–strain responses of the five sensors. As shown in Figure 5d, the five devices exhibited comparable response trajectories, and the pointwise mean and SD were calculated over their common strain range. The corresponding GF values are summarized in Figure 5e, yielding a mean GF of (1.28 ± 0.11) × 10^5^ and a between‐sensor CV of 8.24% (*n* = 5 sensors). These results demonstrate that the standardized fabrication procedure provides reproducible out‐of‐plane crack depths and consistent electromechanical responses across independently fabricated devices.

**FIGURE 6:**
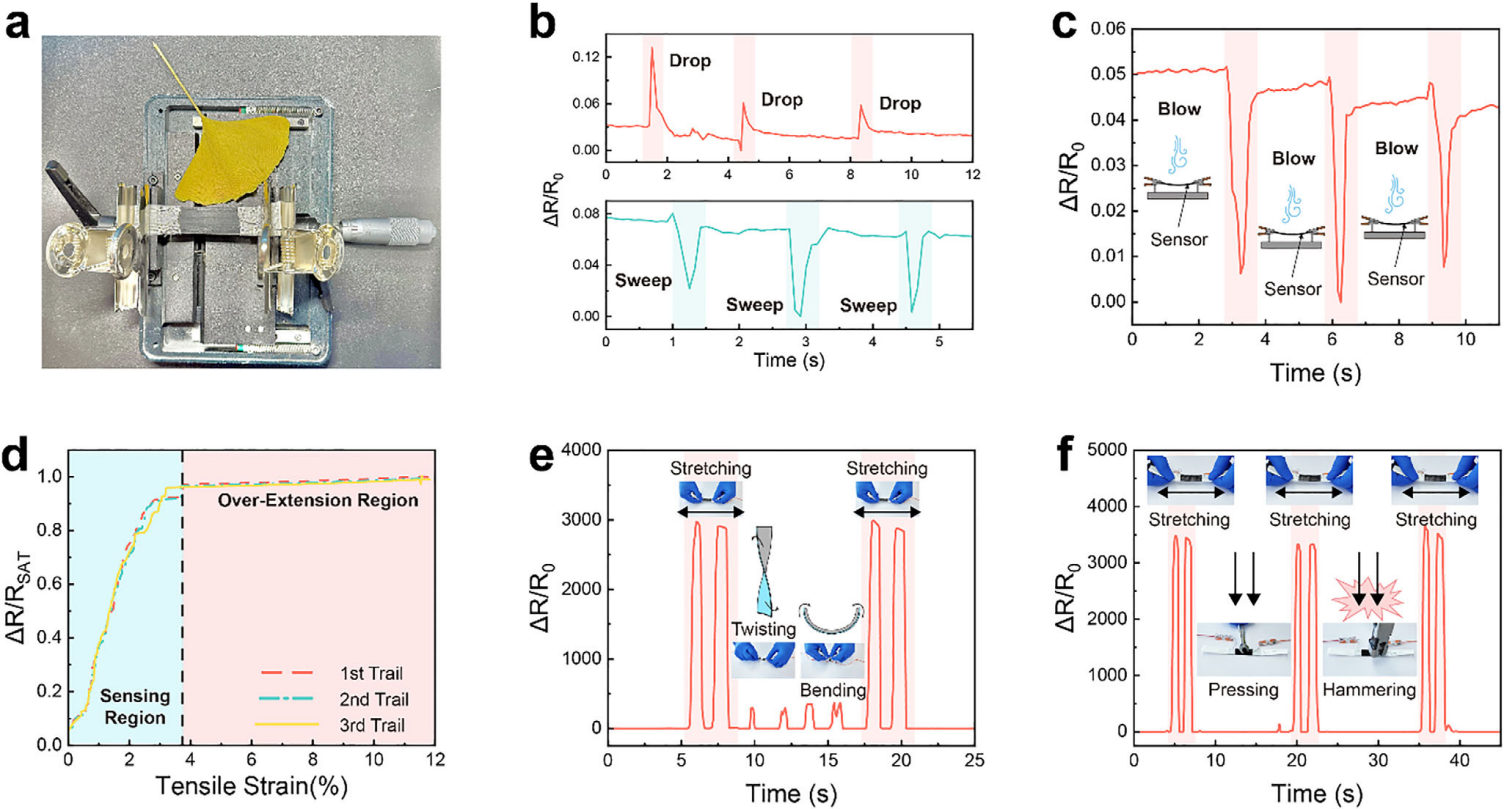
Sensitivity testing and reliability evaluation under complex operating conditions. (a) Photograph of the leaf and sensor suspended in a tensile test setup. (b) Real‐time transient responses of the sensor to falling leaves (top) and gentle brushing (bottom), demonstrating an extremely low detection limit. (c) Non‐contact sensing of weak airflow (air blowing). (d) Overrange testing: Resistance response under strain exceeding the linear limit, showing consistent recovery trajectories. (e) Selective response testing: The sensor exhibits a strong response to tension but is insensitive to torsion and bending, ensuring signal reliability. (f) Response stability under extreme mechanical loads (compression, hammering).

Figure 6 demonstrates the detection limits and robustness of this sensor under extreme operating conditions. By suspending the sensor in mid‐air using a simple tensioning device (Figure 6a), we tested its ability to detect subtle disturbances. As shown in Figure 6b, the sensor can capture transient signals generated by falling leaves making contact with it or by slight brushings in real time. Furthermore, even minor fluctuations in airflow (such as a puff of air, Figure 6c) can induce significant changes in resistance. This demonstrates that the sensor possesses extremely high signal gain and an excellent SNR, making it well‐suited for non‐contact sensing.

In addition, to address potential unexpected overloads that the sensor might encounter, we conducted out‐of‐range tests (Figure 6d). When the strain exceeded its linear limit, although the sensor entered a saturated state, its resistance response did not experience an open circuit. Furthermore, after the stress was released and the sensor was reloaded with tensile stress, the resistance curves overlapped with one another, showing no significant changes. This soft failure characteristic significantly enhances the safety margin of the device in complex real‐world applications.

In practical wearable applications, multimodal deformation often leads to signal coupling. Selective testing using Video S1 demonstrates that the sensor exhibits an extremely high response to axial tensile stress while displaying natural common‐mode rejection characteristics against torsion and bending. Figure 6e illustrates the sensor's resistance response under different strains. This tensile‐specific response ensures signal reliability during the monitoring of complex human movements. Additionally, to evaluate the sensor's survivability in harsh industrial or field environments, we conducted extreme stress tests, as shown in Video S1. Under impact loads such as intense compression and repeated hammering, the sensor's resistive response continued to exhibit rapid recovery and waveform integrity (Figure 6f), demonstrating that the heterogeneous encapsulation structure provides inherent protection against impact‐induced deformation.

### 2.5 Multi‐scale Physiological Monitoring and Remote Human‐Machine Interaction

#### 2.5.1 High‐Fidelity Capture of Whole‐Body Multimodal Physiological Signals

**FIGURE 7:**
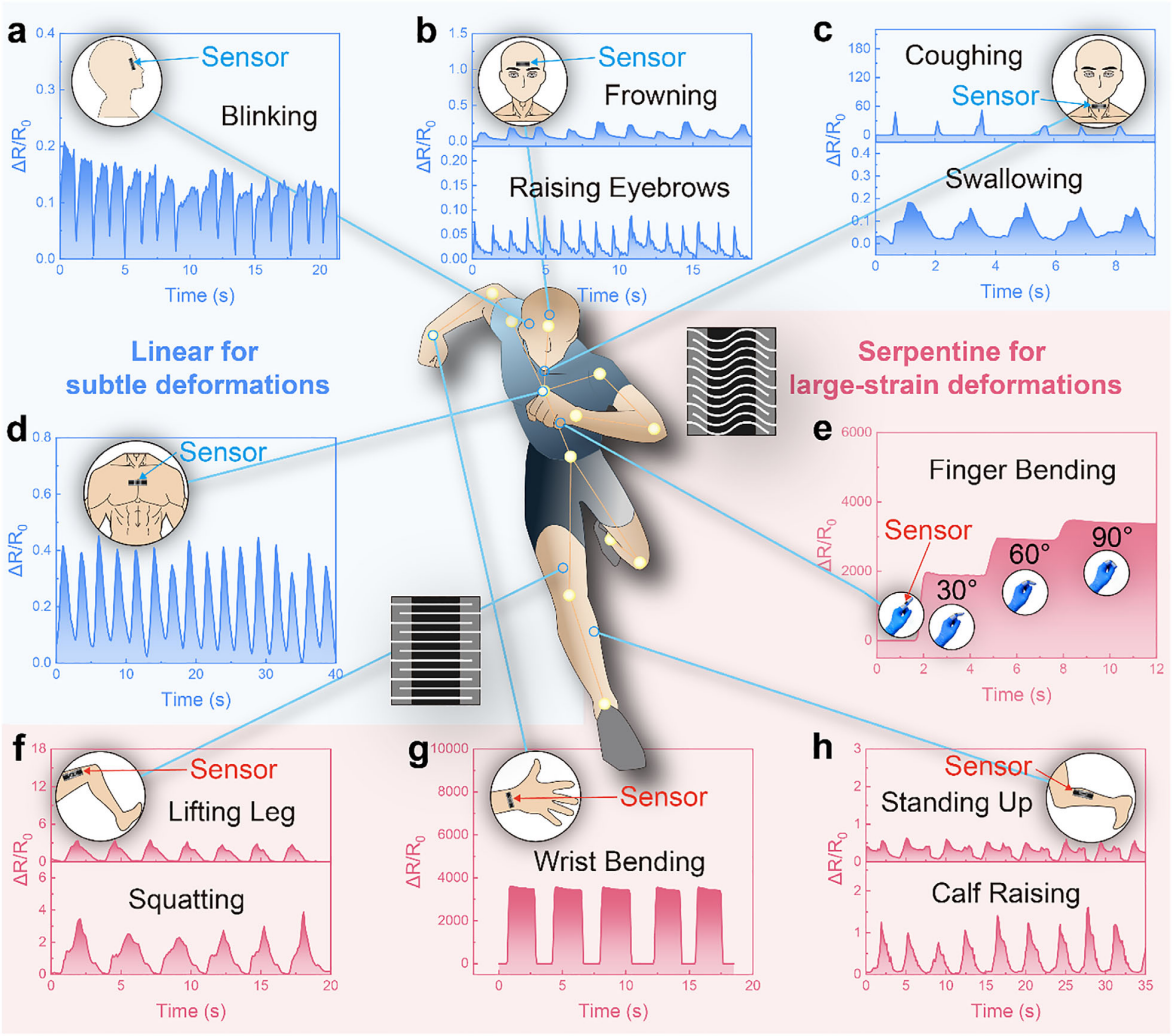
Real‐time monitoring of full‐body physiological features and movement states. Center: Diagram showing the distribution of body sensors. (a–d) Detection of subtle physiological signals using linear crack sensors: (a) eyebrow ridge: blinking, (b) glabella: frowning and eyebrow raising, (c) throat: coughing and swallowing, (d) mid‐chest: respiration monitoring. (e–h) Large‐scale motion detection using serpentine crack sensors: (e) finger flexion at different angles, (f) rectus femoris muscle movement: leg lifting, squatting, (g) wrist bending, (h) gastrocnemius muscle movement: standing up/sitting down, heel raising.

Thanks to the ultra‐high GF and exceptional SNR provided by its heterogeneous structure, this sensor demonstrates exceptional versatility in full‐body motion monitoring (Figure 7). As shown in Figure 7a–d, by attaching linear crack sensors to the corners of the eyes, the forehead, and the neck, it is possible to capture subtle muscle movements such as blinking, frowning, swallowing, and breathing with extremely high fidelity. Using serpentine crack sensors with a wide linear range, we performed real‐time tracking of complex human limb movements (Figure 7e–h). Whether tracking continuous finger flexion, wrist flexion, standing/sitting movements of the lower leg, or high‐intensity squats of the thigh, the sensor demonstrated excellent signal repeatability and dynamic tracking capabilities without noticeable signal lag. This full‐scale coverage, ranging from micrometer‐level skin deformation to centimeter‐level joint displacement, demonstrates the sensor's immense potential in smart healthcare and rehabilitation engineering.

#### 2.5.2 High‐Fidelity HMI System for Robotic Arm Teleoperation

**FIGURE 8:**
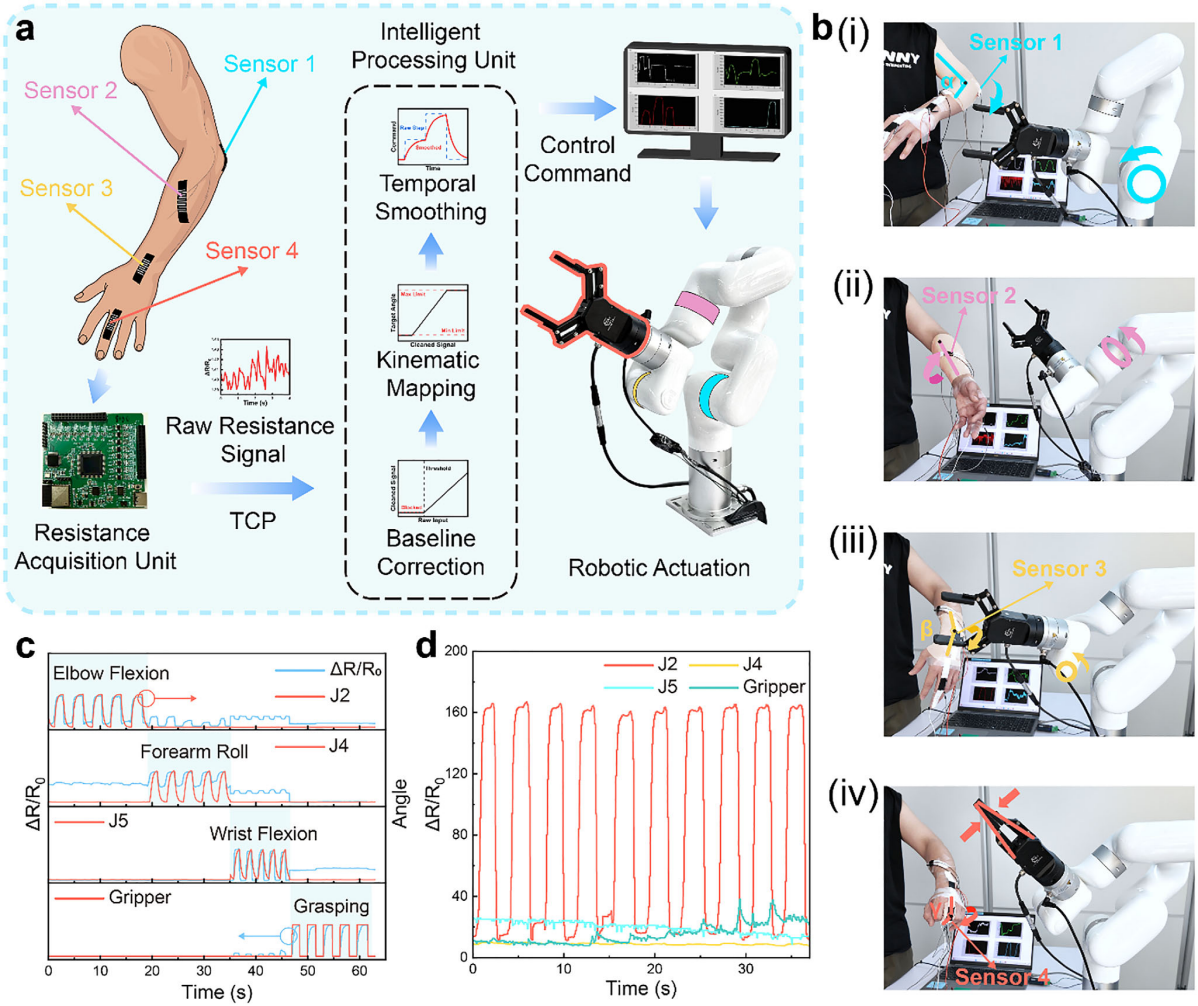
High‐fidelity HMI system for robotic arm teleoperation. (a) Schematic of the four‐channel multi‐joint teleoperation system using a 6‐DOF UFACTORY xArm platform; the elbow, forearm, wrist, and finger sensing channels are mapped to J2, J4, J5, and the gripper, respectively. (b) Photographic sequence showing the robotic arm performing synchronized joint movements and gripper actuation in real time. (c) Real‐time multi‐channel sequential control: Resistance curves from four sensors corresponding to the sequential actuation of the elbow, forearm, wrist, and gripper. (d) Signal decoupling and anti‐interference verification: Demonstrates the precise recognition of target actions by specific sensors during vigorous limb movements.

To further validate the sensor's system‐level integration capabilities, we constructed a multi‐joint teleoperation system using a six‐degree‐of‐freedom (6‐DOF) UFACTORY xArm platform, together with a four‐channel wearable sensor system and a signal‐processing unit (Figure 8a). In the present demonstration, four sensors attached to the user's elbow, forearm, wrist, and fingers acquired independent resistance signals that were mapped to J2, J4, J5, and the gripper, respectively (Table S1). After data processing, this information is converted into motion commands and transmitted to the robotic arm for remote operation [57]. Figure S9 illustrates the relationship between changes in sensor resistance and the robotic arm's angle and gripper position. As shown in the figure, the trends in both variables closely align. Video S2 demonstrates the real‐time interaction between human arm movements and the robotic arm, including independent movements of different joints as well as simultaneous multi‐joint movements of the entire arm. The sequence of photographs (Figure 8b) demonstrates how the robotic arm performs complex joint movements in real time, synchronized by sensor input. Thanks to the sensors’ high linearity and fast response time, the robotic arm's end‐effector can precisely replicate the human operator's intended movements.

As shown in Figure 8c, the sensor array enables the synchronous transmission of multi‐channel time‐series signals, with different body parts corresponding to multiple joints of the robotic arm. The most challenging aspect of the experiment was the decoupling of motion signals. As shown in Figure 8d, when the user performs vigorous elbow movements, the corresponding channel (J2) exhibits significant changes in resistance, while the adjacent wrist sensor demonstrates extremely low crosstalk. This excellent signal decoupling is attributed to the sensors’ specific response to axial stretching, ensuring precision in complex motion control.

This high‐fidelity interaction experience not only validates the reliability of this heterogeneous sensor structure in high‐performance HMI applications but also lays a solid experimental foundation for future applications in telemedicine, hazardous environment operations, and soft robotic skins.

## 3 Conclusion

In summary, we report a high‐performance flexible strain sensor utilizing a bilayer cladding architecture with a U‐shaped cross‐section and deterministic crack engineering. By precisely controlling the conductivity gradient within the conductive sensing layer and employing a strain‐dependent path switching (SDPS) mechanism, we achieved an unprecedented gauge factor of 301,799. Additionally, by controlling the crack and various geometric parameters, we achieved task‐oriented adjustment of sensor performance. For instance, linear crack topologies are suitable for detecting minute deformations, while serpentine crack topologies are suitable for detecting larger deformations. The serpentine crack topology introduces geometric elastic potential energy, which effectively mitigates electromechanical hysteresis and ensures soft failure resilience under extreme impacts. The sensor's exceptional SNR supports high‐fidelity monitoring, ranging from subtle physiological oscillations to complex HMI teleoperation, demonstrating its robustness in precision medicine and synchronous robotic control. This combination of material heterogeneity and geometric structural variation provides a transformative paradigm for overcoming performance bottlenecks in flexible electronic devices, offering both theoretical and hardware foundations for large‐area electronic skin and intelligent multimodal sensing systems [58].

## 4 Experimental Section

### 4.1 Materials

Multi‐walled carbon nanotubes (MWCNTs, outer diameter: 10–20 nm, length: 10–30 nm, purity >95%) were purchased from Beijing Mreda Technology Co., Ltd. Polydimethylsiloxane (PDMS, Sylgard 184) was purchased from Specialty Industrial Adhesives, Inc. Isopropyl alcohol (IPA) and anhydrous ethanol were purchased from Beijing Mreda Technology Co., Ltd. All chemical reagents were of analytical grade and required no further purification before use. Sensors were fabricated using custom‐made grooved polytetrafluoroethylene (PTFE) molds; transparent tape was used to create a U‐shaped structure and determine the width of the central low‐resistance region.

### 4.2 Preparation of Heterogeneous Sensing Composites

CNT/PDMS slurries with different mass fractions were prepared using the solution mixing method. A predetermined mass of MWCNTs was added to isopropyl alcohol (mass ratio of MWCNTs to IPA of 1:50), followed by the addition of the PDMS masterbatch. The mixture was stirred continuously at 60°C for 2 h to evaporate the solvent. After the solvent was completely removed, a curing agent was added (mass ratio of base to curing agent of 10:1), and the mixture was stirred until homogeneous, yielding conductive pastes with 2 wt.% (high‐resistance shell) and 8 wt.% (low‐resistance core) concentrations.

### 4.3 Fabrication of the Biomimetic Heterogeneous Sensor

A deterministic structure was constructed using step‐by‐step casting and mechanical microfabrication techniques. A central low‐resistance region was separated in a PTFE grooved mold using transparent tape, and the 8 wt.% paste was filled into the PTFE mold. A blade coating process was used to ensure a smooth surface. The assembly was precured at 80°C for 1 h to complete curing and enhance electrical conductivity. The transparent tape was then removed from the mold, and the 2 wt.% slurry was poured around the periphery of the precured core to form a complete nested structure. The entire assembly is then cured at 80°C for 1 h. The PDMS base material and curing agent are thoroughly mixed, degassed under vacuum, and injected into the PTFE mold with the cured sensing layer, followed by curing at 80°C for 3 h. After full curing, the assembly is peeled out of the mold.

Predefined crack arrays were introduced using a Silhouette CAMEO 5 desktop cutting machine (Silhouette America, Inc., USA) equipped with an AutoBlade mounted in Tool 1. The CNT/PDMS specimens were placed flat on a 12 × 12 in. (304.8 × 304.8 mm) cutting mat with the cutting surface facing upward and were secured using four strips of adhesive tape positioned along the four edges outside the cutting region. In Silhouette Studio, the blade‐depth, force, and cutting‐speed settings were set to 6, 15, and 3, respectively, and each programmed cutting path was executed in a single pass. The nominal crack length, pitch, and topology were defined by the digital cutting design. Each cutting path was extended slightly beyond the nominal crack endpoints to ensure complete cutting across the target conductive region, and the excess terminal portions were removed during subsequent specimen trimming. Finally, conductive silver wires were attached to both ends of the sensing layer as electrical leads.

### 4.4 Characterization and Measurements

A field‐emission scanning electron microscope (ICCAS8230) was used to observe the cross‐sectional morphology of the sensor and the bonding condition at the heterogeneous interfaces. The electromechanical performance testing system consists of the following components: A Mark‐10 Model F105 materials testing machine provides precise recording of tensile displacement and force values. A 32‐channel parallel resistance acquisition system was developed in‐house.

### 4.5 Finite Element Analysis

3D coupled electromechanical simulations were performed using COMSOL Multiphysics 6.4 by combining the Solid Mechanics and Electric Currents interfaces. For the geometry‐dependent simulations, the low‐resistance core width D, predefined crack length Lg, crack pitch P, or crack topology was varied according to the corresponding parametric study, while all other material, electrical, interfacial, and loading parameters were held constant. The predefined crack paths were prescribed by the deterministic cut geometry, and progressive separation and electrical contact loss were calculated along these interfaces. One longitudinal end was fixed, while displacement was applied to the opposite end. A potential difference of 1 V was imposed between the two longitudinal ends. Additional model details are provided in Note S1.

### 4.6 Human Physiological Signal Sensing

Flexible sensors were integrated onto breathable medical adhesive tape and applied to specific body parts of the participants. Linear strain sensors were attached to the inner corners of the eyes, eyebrows, Adam's apple, and chest to monitor blinking, swallowing, and respiratory signals. Curved strain sensors were affixed to the fingers, elbows, gastrocnemius muscles, and rectus femoris muscles. Participants performed predetermined movement sequences (such as continuous bending and squatting), and a 32‐channel acquisition system captured the motion‐specific impedance spectra of each body part in real time.

### 4.7 Integration of Robotic Teleoperation System

A real‐time human‐machine interface (HMI) control system based on impedance mapping was developed. The sensor array uses a 32‐channel acquisition system to collect real‐time resistance change data. Read impedance dataImpedance data were read via the serial protocol at a frequency of 100 Hz. Utilizing the common‐mode rejection characteristics provided by the anisotropic structure, motion signals in different dimensions were linearly decoupled. The normalized resistance‐change signals (ΔR/R_0_) from sensors attached to the elbow, forearm, wrist, and finger were linearly mapped to the commanded angles of J2, J4, and J5 and the commanded gripper position of a 6‐DOF UFACTORY xArm platform, respectively, as summarized in Table S1. The operator remotely controls the robotic arm to perform complex movements and tasks such as finger opening and closing through coordinated arm and finger movements. The system's real‐time performance and spatial consistency are evaluated by comparing the response latency between the operator's motion commands and the robotic arm's end‐effector.

### 4.8 Ethical Statement for Human Subject Testing

All experiments involving human subjects were performed in accordance with the relevant institutional guidelines and were reviewed and approved by the Committee on Biological and Medical Ethics, Beihang University (approval No. BM20230120). Written informed consent was obtained from all participants prior to participation.

## Author Contributions

**Qi Wu**: methodology, data curation, investigation, validation, writing – original draft, visualization, formal analysis, software. **Tiantong Wang**: conceptualization, methodology, writing – review and editing, investigation, supervision. **Yinhao Wang**: investigation. **Yanggang Feng**: resources, data curation. **Yan Huang**: conceptualization, investigation, supervision, funding acquisition, project administration, writing – review and editing.

## Funding

This work is supported by the Beijing Institute of Technology Research Funds for High‐Level Talents. This work is also supported by the China Postdoctoral Science Foundation under Grant Number 2024M764126 and the Postdoctoral Fellowship Program of CPSF under Grant Number GZC20242173.

## Conflicts of Interest

The authors declare no conflicts of interest.

## Supporting information




**Supporting File 1**: advs77979‐sup‐0001‐SuppMat.docx.

**Supporting File 2**: advs77979‐sup‐0002‐VideoS1.mp4.

**Supporting File 3**: advs77979‐sup‐0003‐VideoS2.mp4.

## Data Availability

The data that support the findings of this study are available from the corresponding author upon reasonable request.
