# Supplementary material for: Bio‐Inspired Gradient Heterogeneous Architectures for Ultrasensitive and Robust Strain Sensors via Programmable Crack Engineering
Source: Adv Sci (Weinh). 2026 Sep 27:e77979. Online ahead of print. doi: 10.1002/advs.77979 (PMC13616321; doi:10.1002/advs.77979)
Supplement: Supplementary file 1 [file ADVS-9999-e77979-s002.docx]

**Supporting Information**

**Bio-inspired Gradient Heterogeneous Architectures for Ultrasensitive and Robust Strain Sensors via Programmable Crack Engineering**

Qi Wu^1^, Tiantong Wang^1^, Yinhao Wang^1^, Yanggang Feng^2^, Yan Huang^1^*

^1^Key Laboratory of Biomimetic Robots and Systems of Ministry of Education, School of Mechatronics Engineering, Beijing Institute of Technology, Beijing 100081, China

^2^School of Mechanical Engineering and Automation, Beihang University, Beijing 100191, China

**Supplementary Note S1. Finite Element Analysis**

A family of three-dimensional coupled electromechanical models was constructed using COMSOL Multiphysics 6.4. For the parametric analyses, D, L_g_, P, or crack topology was varied according to the corresponding simulation series, while the remaining geometry, material properties, interface parameters, electrical conditions, and loading conditions were held constant.

The model had overall dimensions of 69 × 16 × 2 mm^3^, with an effective gauge length of L_0_=60 mm. Nominal tensile strain was calculated as

$$\varepsilon=\frac{u}{L_{0}}$$

where u is the applied displacement. One longitudinal end was fixed, and displacement was applied to the opposite end. A stationary displacement sweep from 0 to 21 mm was performed, corresponding to nominal strains from 0% to 35%.

The solid domains were described using a Neo-Hookean constitutive model. The electrical conductivities of the low- and high-resistance regions were 79.58 Sm^−1^ and 6.253 × 10−3 Sm^−1^, respectively. A potential difference of V = 1 V was applied between the two longitudinal ends.

The spatial locations of the crack interfaces were determined by the predefined cut geometry. Progressive separation along these interfaces was represented using a linear cohesive traction–separation relation. The electrical contact conductance was coupled to the cohesive damage variable according to

$$g_{c}\left( d \right)=g_{0}f\left( d \right)$$

where g_c_ is the strain-dependent electrical contact conductance, g_0_ = 1.2 × 10^3^ Sm^−2^ is the initial contact conductance, d is the cohesive damage variable, and f(d) is a monotonically decreasing function. Here, d = 0 represents the initially contacted interface and increasing d represents progressive interface separation and electrical contact loss.

The device resistance at each applied displacement was obtained from the terminal current:

$$R\left( u \right)=\frac{V}{I\left( u \right)}$$

The relative resistance change was calculated using the resistance obtained from the undeformed model:

$$\frac{\Delta R}{R_{0}}=\frac{R\left( u \right)-R\left( 0 \right)}{R\left( 0 \right)}$$

For comparison across different loading stages, the current-density magnitude and von Mises stress were normalized as

$$J^{*}=\frac{\left| \mathbf{J} \right|}{J_{ref}}$$

and

$$\sigma^{*}=\frac{\sigma_{vM}}{\sigma_{ref}}$$

where J_ref_ and σ_ref_ are common reference values used for all loading stages displayed in the same figure. The model was discretized using mapped surface elements and swept hexahedral volume elements, with local refinement near the predefined crack interfaces.

**Table S1.** Channel to output mappings used in the multi-joint teleoperation demonstration.

| Channel | Sensor placement / motion | Controlled output | Output range |
| --- | --- | --- | --- |
| 1 | Elbow / flexion | J2 angle | -45° to -15° |
| 2 | Forearm / rotation | J4 angle | -35° to +35° |
| 3 | Wrist / flexion | J5 angle | -90° to 0° |
| 4 | Finger / grasping | Gripper position | 0 (open) to 9000 (closed) device units |

**Table S2.** Exploratory Type III two-factor ANOVA of the device-level mean GF and linear sensing range after log10 transformation.

| Design | Response | Effect | F(df1, df2) | p |
| --- | --- | --- | --- | --- |
| D–L_g_ | GF | D | F(2,10)=5.75 | 0.0217 |
| D–L_g_ | GF | L_g_ | F(2,10)=6.00 | 0.0194 |
| D–L_g_ | GF | D×L_g_ | F(4,10)=2.00 | 0.1701 |
| D–L_g_ | Linear range | D | F(2,10)=2.44 | 0.1367 |
| D–L_g_ | Linear range | L_g_ | F(2,10)=1.13 | 0.3613 |
| D–L_g_ | Linear range | D×L_g_ | F(4,10)=2.18 | 0.1445 |
| P–geometry | GF | P | F(2,10)=0.84 | 0.4610 |
| P–geometry | GF | Geometry | F(2,10)=56.87 | <0.0001 |
| P–geometry | GF | P×Geometry | F(4,10)=20.66 | <0.0001 |
| P–geometry | Linear range | P | F(2,10)=19.22 | 0.0004 |
| P–geometry | Linear range | Geometry | F(2,10)=40.73 | <0.0001 |
| P–geometry | Linear range | P×Geometry | F(4,10)=3.61 | 0.0454 |

Device-level mean values were used as independent observations. Each design contained 19 observations across nine parameter combinations, with n = 2 per condition except for the shared reference condition (D = 7 mm, L_g_ = 14 mm, P = 1.2 mm, straight crack), where n = 3. Fixed-effects models were fitted to log10-transformed responses using Type III sums of squares and sum-to-zero contrasts. The analyses were considered exploratory because of the limited replication per condition.

**Figure S1.** The specific fabrication process for the sensor. First, use transparent tape to mark out the area for the low-resistance sensitive layer on the PTFE mold. Mix CNT, PDMS prepolymer, and IPA in different mass ratios. After heating to evaporate the IPA, add the PDMS curing agent and mix thoroughly. Apply the low-resistance mixture by scraping it into the gaps between the tape strips on the PTFE mold, then heat at 80°C for one hour. Remove the transparent tape to form the low-resistance sensitive layer. Subsequently, the high-resistance mixture is spread onto the PTFE mold, and after heating at 80 °C for one hour, the entire sensitive layer area is obtained. Mix the PDMS prepolymer and curing agent in a 10:1 ratio until uniform. After degassing under vacuum, pour the mixture into a PTFE mold. Heat at 80 °C for 3 hours until fully cured, then demold. Use a mechanical cutting machine to cut a crack structure on the surface of the sensitive layer.

**Figure S2.** Microscopic SEM images of the sensor. a) Cross-sectional view of the sensor showing the interface between the double-layer sensitive layer and the elastic substrate. b) Cross-sectional view of the sensor showing the double-layer U-shaped encapsulation structure of the sensitive layer. c) High-magnification top view of the interior of the slit in the low-resistance section of the sensor. d) High-magnification top view of the slit in the high-resistance section of the sensor.

**Figure S3.** Trends in the resistivity of CNT/PDMS composites as a function of CNT mass fraction.

**Figure S4.** Geometric definitions and programmed cutting paths used for deterministic crack design. a) Plan-view schematic of the straight-crack sensor defining the central low-resistance-core width (D), projected crack length (L_g_), crack pitch (P), and straight crack path (λ = 1). b) Programmed cutter trajectories for the (i) large-period and (ii) small-period wavy cracks. The solid orange portions represent the crack paths retained within the prescribed projected interval L_g_, whereas the dashed orange portions denote sacrificial extensions of the programmed cutting trajectory outside the retained region. These external portions were removed during cutting and trimming so that all devices maintained the same transverse dimension and projected crack length. The gray dashed line denotes the straight reference centerline. A is the lateral amplitude, Λ is the spatial period measured between equivalent points along the wave, and the tortuosity factor is defined as λ = L_path_/L_g_, where L_path_ is the actual length of the retained crack path.

**Figure S5.** Device-level interaction plots for the D × L_g_ full-factorial experiment. (a) GF and (b) linear sensing range as functions of L_g_ at D = 4, 7, 10 mm. Faint symbols represent individual devices, and connected symbols represent condition means.

**Figure S6.** Results of the Cyclic Stability Test for Curved Crack Sensors.

**Figure S7.** Low-magnification SEM characterization of predefined cracks across five independently fabricated sensors. Five cracks (C1–C5) were randomly selected from each of five sensors (S1–S5) fabricated using identical procedures and observed at 900× magnification in the unloaded state. The images show the formation and initial closure morphology of the mechanically predefined cracks. Scale bars: 50 μm.

**Figure S8.** High-magnification SEM characterization of representative predefined cracks from five independently fabricated sensors. The C3 crack from each sensor was examined at 7000× magnification: a) S1, b) S2, c) S3, d) S4, and e) S5. The images reveal the narrow and tightly reclosed crack morphology in the unloaded state, for which a reproducible in-plane crack-tip boundary and opening angle could not be reliably extracted. Scale bars: 5 μm.

**Figure S9.** The relationship between changes in sensor resistance and the robotic arm's angle and gripper position.
